# Tracing ovarian cancer research in Morocco: A bibliometric analysis

**DOI:** 10.1016/j.gore.2021.100777

**Published:** 2021-05-07

**Authors:** Khalid El Bairi, Ouissam Al Jarroudi, Said Afqir

**Affiliations:** a Department of Medical Oncology, Mohammed VI University Hospital, Oujda, Morocco; b Faculty of Medicine and Pharmacy, Mohammed I^st^ University, Oujda, Morocco

**Keywords:** Ovarian cancer, Cancer research, Bibliometric, Scientometric, Morocco, BRCA, Breast cancer susceptibility gene, GDP, gross domestic product, IARC, International Agency for Research on Cancer, OC, ovarian cancer, OCAC, The Ovarian Cancer Association Consortium, US, United States, WoS, Web of Science

## Abstract

**Background:**

The burden of ovarian cancer (OC) in low-income countries continues to increase annually. This gynecological cancer, known for its poor survival outcomes, has not attracted much interest in medical research as compared to other women’s malignancies such as breast cancer. This bibliometric study was conducted to better depict the global map and the future directions of scientific productivity in the area of OC research in Morocco.

**Methods:**

Publication trends on OC were retrospectively analyzed using a number of bibliometric parameters based on the Pubmed database and other resources.

**Results:**

During the time period (1900–2018), a total number of 74 publications responding to the inclusion criteria were found and incorporated in the bibliometric analysis. This was dominated by case reports and case series on rare ovarian tumors (n = 60). In the core cluster, only 10 original studies and 3 reviews on OC were published by Moroccan researchers. After full-text appraisal for study population, only two clinical original articles included OC patients. The other clinical studies included breast cancer patients only or were suggestive of inherited OC. In addition, 3 preclinical *in vitro* studies were found during the literature search. The majority of these publications were covered by Pubmed and Web of Science core collection and all published in English language. The H-index of top 10 Moroccan scientists in this area didn’t exceed 10. Importantly, research and review articles were frequently published in influential journals. However, the number of publications as compared to other African countries was very low. Moreover, a similar trend in terms of article per each newly diagnosed OC case, GDP per capita and per million was also noticed. For gender distribution, female scientists were first authors in the majority of these papers but less represented as leading last authors. In the complementary cluster of other article types on rare ovarian tumors, 70% of the items were published in French and approximately 60% were indexed on Pubmed. During the last five years, a marked acceleration of publishing this research category with little impact in the evidence-based practice was noticed.

**Conclusions:**

This research area in gynecologic oncology seems to be neglected and needs to be prioritized in future research projects in Morocco particularly given the aggressive behavior of this women’s cancer and the few available therapeutic options. There is an unmet need for studies on OC in all fields particularly epidemiology, clinic-pathological characteristics, and survival outcomes.

## Highlights

• Research on ovarian cancer in Morocco seems to be neglected.
• Nearly all publications were dominated by case reports and case series on rare ovarian tumors.
• There is an urgent need for studies on ovarian cancer in all fields.

## 1 Introduction

Historically, “*Bibliometrics*” was first introduced into the literature by the Belgian librarian Paul Otlet in his book “*Traité de Documentation*” in 1934 (Rousseau, 2014). The author defined this new term as “*the measurement of all aspects related to the publication and reading of books and documents*” (Rousseau, 2014, Otlet, 1934). Since then, an important number of bibliometric reports to analyze the structure of the published literature in various areas in science were published, particularly in medicine with more than 12,400 articles found on Pubmed (as of 11–07-2020) (Kokol et al., 2020, pubmed.ncbi.nlm.nih.gov, 2020). Bibliometrics plays a significant role in the quantitative and qualitative assessment of research landscapes of particular fields (Haustein and Larivière, 2015, Belter, 2015). This may considerably impact research projects, guide the design of future studies, and boost the national contribution in scientific productivity to achieve superior international visibility. Moreover, bibliometric investigations have also a powerful role in governmental policies and strategies to improve and support decision-making, disease control, and patients’ care (Ismail et al., 2012, Thompson and Walker, 2015).

Ovarian cancer (OC) is still a leading cause of high rates of mortality from gynecological cancers (Webb and Jordan, 2017). OC is the 7th most common cancer and the 8th in terms of mortality among women worldwide (Webb and Jordan, 2017, Coburn et al., 2017, Momenimovahed et al., 2019). According to the latest updates of the GLOBOCAN database (available at: https://gco.iarc.fr/), OC in Morocco is the third gynecological cancer in terms of incidence and is ranked 15 with 1222 new cases for both sexes in 2020 for all cancer sites. OC has a 5-years prevalence of 15.81 per 100,000 and is also the third gynecological cancer in terms of mortality. Despite recent advances in therapy, OC has 5-year relative survival below 45% (Webb and Jordan, 2017). This is mainly due to the diagnosis in advanced stages and resistance to the standard platinum-based chemotherapy. The marked poor prognostic outcomes observed in this women’s cancer have raised awareness toward advancing clinical and translational research to uncover the mechanisms of this aggressive disease, develop early detection strategies, and find additional therapeutics beyond platinum-based combinations. Promisingly, the published research related to OC is continuously increasing (Brüggmann et al., 2017). However, this trend is concentrated in high-income countries as compared to lower-resources nations (Brüggmann et al., 2017). Previously, only two bibliometric studies have provided a mapping of the research architecture of OC research in Turkey (Guler et al., 2013) and globally (Brüggmann et al., 2017). Unfortunately, almost no visible role in the productivity of African OC publications in the scientific community has been noticed despite the high burden of this disease in their population (Brüggmann et al., 2017).

Our report aims to provide a global overview of OC research in Morocco. The study period was fixed between 2018 and other previous years before we started our projects to develop research on OC in our setting. Hypothetically, this is anticipated to examine the need for additional research on specific topics in this area. To the best of our knowledge, this is the third bibliometric report on OC research worldwide and the first to be conducted in Morocco.

## 2 Methods

### 2.1 Search strategy

**Fig. 1:**
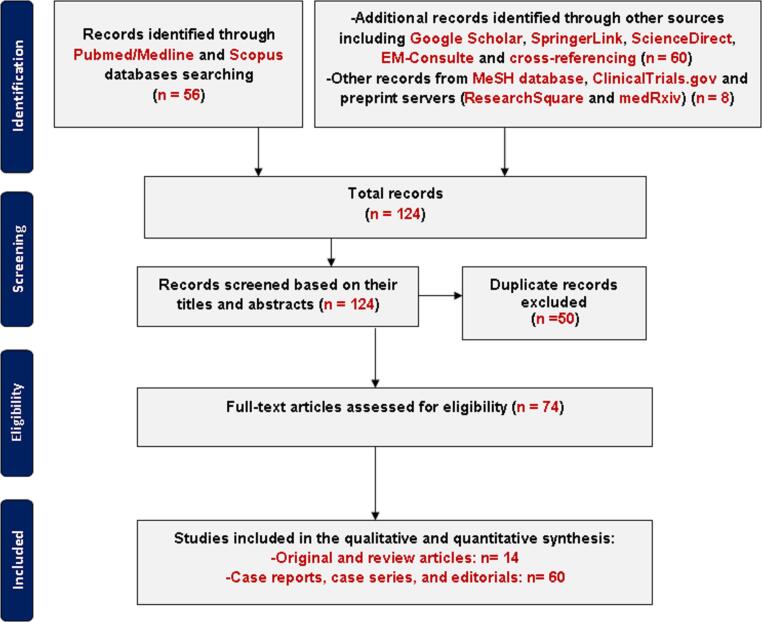
Flow chart of article selection.

We used abstracting/indexing engines and full-text databases to find published articles on OC by Moroccan scientists retrospectively. Additionally, other sources including cross-referencing and Google Scholar were checked to find more publications. The search strategy is described as follows: advanced search on Pubmed/Medline (National Center for Biotechnology Information), which covers most of the medical journals, and Scopus (Elsevier®) using the following combinations of keywords: “ovarian carcinoma”, OR “cancer of the ovary”, OR “ovarian malignancy”, OR “ovarian tumor”, OR “ovarian neoplasm” AND “Morocco”. The MeSH database was also searched: (“ovarian Neoplasms”[Mesh]) AND “Morocco”[Mesh]. Moreover, additional searches based on cross-referencing, SpringerLink (Springer Nature®), and Google Scholar using the same previous keywords were screened and provided other papers not covered by Pubmed. To limit language bias, EM-Consulte (Elsevier Masson®) and ScienceDirect (Elsevier®) were selected to cover the Francophone literature. Journal Citation Reports^TM^ 2020 (Clarivate Analytics) was used to find updated journal impact factors. A 1900 (01–01) to 2018 (30–12) analysis of studies that focused on ovarian malignancies was used to find relevant articles. The period selection was chosen to fit the start of our project to develop research on OC in 2018. Selected articles were preliminary checked for eligibility based on their titles and abstracts, and then fully verified for OC patients’ inclusion in their study population. Only peer-reviewed and published papers during the period 1900–2018 were selected (Fig. 1). Ongoing studies and pre-prints from ClinicalTrials.gov, ResearchSquare, and medRxiv were excluded. In an attempt for comparison with other cancer types and research outputs in other countries, only Pubmed search was used. Bibliographic searching based on these criteria was run independently twice.

The GLOBOCAN official website (Cancer Today-IARC: available at: https://gco.iarc.fr/today/online-analysis-map) was used to collect the absolute numbers of OC age-standardized incidence, and the crude rate which is calculated by dividing the number of new cases for a specific cancer observed during a given time period by the corresponding number of person years in the population at risk (usually expressed as an annual rate per 100,000 persons at risk) (www-dep.iarc.fr, 2020). We then calculated the ratio of country-specific articles per each new OC case in Morocco as previously described (Brüggmann et al., 2017) and per million inhabitants. Moreover, a socioeconomic quantification of country-specific contributions concerns the economic resources which were investigated based on the gross domestic product (GDP) per capita, and population size using the databases of The World Bank (available at: https://data.worldbank.org/indicator/NY.GDP.PCAP.CD?locations=MA). Finally, we defined international collaboration when at least one author with a foreign affiliation had contributed to the study.

### 2.2 Data analysis and categorization

Data related to: author/year, article title, article type, article language, journal, open access or subscription model, Pubmed indexing, Web of Science (WoS) indexing**,** research field, total number of authors, total number of female authors, male–female ratio, funding and international collaboration (for reviews and original articles only), journal impact factors according to the latest version of Journal Citation Reports 2020, and first authors’ H-index according to Scopus database (as of 15-07-2020), were extracted and summarized in 4 different tables. Firstly, data were extracted manually and analyzed using Microsoft Office Excel 2007 (Microsoft, Redmond, WA, USA) for basic statistics. For a better qualitative assessment, our findings were categorized into two clusters of papers including a cluster of reviews and original articles and an additional cluster of case reports, case series, and editorials for rare ovarian malignancies. This was conducted for a better assessment of article types. Ethical committee approval was not required as the study design is based on available published research. Publications authors were not contacted for further information regarding their published studies.

## 3 Results

### 3.1 Core cluster: research and review articles

**Fig. 2:**
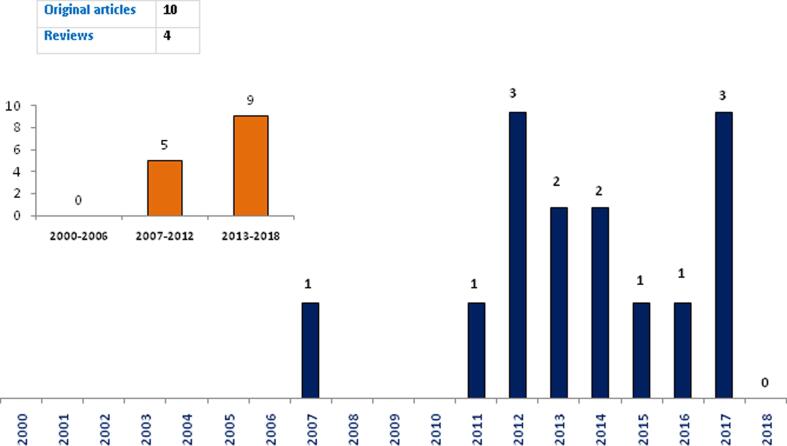
Evolution of number of original and review articles per year.

During the study period, 14 articles encompassing 10 original articles and 4 reviews were included in the selection/verification process (Table 1, Fig. 2). Eight of them were published using the open access model. Only one publication (Amrani et al., 2014) was not indexed on Pubmed. For WoS, two papers published in *Breast Disease* and *Biomedical Engineering Research* (Amrani et al., 2014, Cherbal et al., 2012) were not found on this highly selective database. All of these articles were published in English language and were mostly in the clinical area (11/14) and half of these publications declared receiving funding from national and international organizations. Regarding international collaboration, 9 of the 14 publications included at least one author from a foreign affiliation, mainly from the European Union (France and Italy). Table 2 shows the evolution of publications by research field and funding. During the period 2000–2010, no article in the clinical area was found. Thereafter, 11 publications were found including 7 original articles and 3 reviews in addition to another review from an Algerian team that described the *BRCA* mutational status in hereditary breast and OCs in the Maghrebian countries by Cherbal et al. (2012). Notably, of these 7 original studies, only two reports had OC patients in their study population (Amrani et al., 2014, Tazzite et al., 2012). Similarly, fundamental research papers were rarely observed. Three *in vitro* studies that investigated the pharmacological activities of natural and synthetic compounds on OC cells were found during this period (Brüggmann et al., 2017, Abbassi et al., 2012, Ait M'barek et al., 2007).

**Table 1:** Summary of reviews and original articles.

Author/Year	Article title§	Article type	Research field	Study population	Open Access	Journal	Indexing		Article language	Acknowl-edged funding	International collaboration
							Pubmed	WoS			
Laarabi et al. (2017)	High frequency of the recurrent c.1310_1313delAAGA BRCA2 mutation in the North-East of Morocco and implication for hereditary breast–ovarian cancer prevention and control	Original	Clinical	Patients with suggestive inherited breast cancer (BC) and OC	Yes	*BMC Res Notes*	Yes	Yes	English	No	No
El Bairi et al. (2017)	Emerging diagnostic, prognostic and therapeutic biomarkers for ovarian cancer	Review	Clinical	–	No	*Cell Oncol*	Yes	Yes	English	No	Yes
El Bairi et al. (2017)	Prediction of therapy response in ovarian cancer: Where are we now?	Review	Clinical	–	No	*Crit Rev Clin Lab Sci*	Yes	Yes	English	No	Yes
Jouali et al. (2016)	First application of next-generation sequencing in Moroccan breast/ovarian cancer families and report of a novel frameshift mutation of the BRCA1 gene	Original	Clinical	Patients with BC only	Yes	*Oncol Lett*	Yes	Yes	English	No	No
Laraqui et al. (2015)	BRCA genetic screening in Middle Eastern and North African: mutational spectrum and founder BRCA1 mutation (c.798_799delTT) in North African	Review	Clinical	–	Yes	*Dis Markers*	Yes	Yes	English	No	Yes
Amrani et al. (2014)	Immunohistochemical Analysis of WT1, EGFR, E-cadherin, beta-catenin and p53 in 43 Moroccan Epithelial Ovarian Tumours	Original	Clinical	Benign, borderline and invasive epithelial ovarian tumors	Yes	*Biomed. Eng Res*	No	No	English	Yes	Yes
Abbassi et al. (2014)	Synthesis and Antitumor Activity of Some Substituted Indazole Derivatives	Original	Fundamental	–	No	*Arch Pharm*	Yes	Yes	English	Yes	Yes
Sekkate et al. (2013)	Ovarian granulosa cell tumors: a retrospective study of 27 cases and a review of the literature	Original	Clinical	Patients with Granulosa cell tumors	Yes	*World J Surg Oncol*	Yes	Yes	English	No	No
Laraqui et al. (2013)	Mutation screening of the BRCA1 gene in early onset and familial breast/ovarian cancer in Moroccan population	Original	Clinical	BC patients only	Yes	*Int J Med Sci*	Yes	Yes	English	Yes	Yes
Tazzite et al. (2012)	BRCA1 and BRCA2 germline mutations in Moroccan breast/ovarian cancer families: novel mutations and unclassified variants	Original	Clinical	Mostly BC patients with two cases of OC	No	*Gynecol Oncol*	Yes	Yes	English	Yes	Yes
Cherbal et al. (2012)	BRCA1 and BRCA2 germline mutation spectrum in hereditary breast/ovarian cancer families from Maghrebian countries	Review	Clinical	–	No	*Breast Dis*	Yes	No	English	Yes	No
Abbassi et al. (2012)	Synthesis, antiproliferative and apoptotic activities of N-(6(4)-indazolyl)-benzenesulfonamide derivatives as potential anticancer agents	Original	Fundamental	–	No	*Eur J Med Chem*	Yes	Yes	English	Yes	Yes
Laarabi et al. (2011)	Genetic testing and first presymptomatic diagnosis in Moroccan families at high risk for breast/ovarian cancer	Original	Clinical	BC patients only	Yes	*Oncol Lett*	Yes	Yes	English	No	No
Ait M'barek et al. (2007)	Cytotoxic effect of essential oil of thyme (Thymus broussonettii) on the IGR-OV1 tumor cells resistant to chemotherapy	Original	Fundamental	–	Yes	*Braz J Med Biol Res*	Yes	Yes	English	Yes	Yes

§ Article titles were copied as shown in the journals. ^ǂ^Authors from Algeria.

**Table 2:** Evolution of publications (original and review articles) by research field, funding, and gender.

Years	Clinical research papers (N)	Fundamental research papers (N)	Funded publications (N)	Total number of authors	Total number of female authors	Total number of females as first authors	Total number of females as last authors
2007	0	1	1	13	1	0	0
2011	1	0	0	8	3	1	0
2012	2^ǂ^	1	2	19	9	2	1
2013	2	0	1	19	5	1	0
2014	1	1	2	6	3	2	0
2015	1	0	0	12	3	0	0
2016	1	0	0	8	6	1	0
2017	3	0	0	21	9	1	1
Total	11	3	6	106	39	8	2

N = number of articles. ^ǂ^Including one article from Algeria.

Regarding the H-index (Table 3), which is an important parameter to measure the eminence of researchers, the top ten Moroccan researcher’s H-index ranged from 2 (Jouali F and Sekkate S, geneticist and medical oncologist, respectively) to 10 (Amrani M, pathologist). For journal impact factors according to the latest Journal Citation Reports (Clarivate Analytics®), there was a trend in publishing in prestigious journals from respected academic publishers. The highest impact factor was noticed for a preclinical study by Abbassi et al. (2012) (5.572/European Journal of Medicinal Chemistry) (Abbassi et al., 2012), followed by El Bairi et al. (2017) (5.304/Cellular Oncology) (El Bairi et al., 2017), El Bairi et al. (2017) (4.677/Critical Reviews in Clinical Laboratory Sciences) (El Bairi et al., 2017), and Tazzite et al. (2012) (4.623/Gynecologic Oncology) (Tazzite et al., 2012) (details can be found in Table 3).

**Table 3:** Top 10 journals and authors, and their corresponding impact factors and H-Index.

Rank	Journals and their publishers and impact factors^†^	Authors (H-index)^ǂ^
1	-European Journal of Medicinal Chemistry (Elsevier; IF: 5.572)	Amrani M (10)
2	-Cellular Oncology (Springer; IF: 5.304)	Laarabi FZ (8) El Bairi K (8)
3	-Critical Reviews in Clinical Laboratory Sciences (Taylor and Francis; IF: 4.677)	Tazzite A (6)
4	-Gynecologic Oncology (Elsevier; IF: 4.623)	Laraqui A (5) Abbassi N (5) Ait M'barek L (5)
5	-Disease Markers (Hindawi; IF: 2.733)	Jouali F (2) Sekkate S (2)
6	-Archiv der Pharmazie (Wiley; IF: 2.59)	–
7	-International Journal of Medical Sciences (Ivyspring International Publisher; IF: 2.523)	–
8	-Oncology Letters (Spandidos Publications; IF: 2.311)	–
9	- Brazilian Journal of Medical and Biological Research (Associacao Brasileira de Divulgacao Cientifica; IF: 2.023)	–
10	-World Journal of Surgical Oncology (Biomed Central; IF: 1.963)	–

Abbreviations: IF: impact factor. ^†^According to JCR Clarivate Analytics 2020. ^ǂ^ According to Scopus (as of 15/07/2020).

Because we didn’t find any recent bibliometric study on this topic from North African countries, we used Pubmed to map the global landscape of research on OC in general. This pre-screening strategy has several limitations such as the significant overlap with other ovarian tumors particularly rare diseases. From this viewpoint, we aimed to compare Moroccan contributions with other regional countries such as Egypt, Spain, France, Algeria, Tunisia, and some other African countries as shown in Fig. 1 in Supplemental 1. In North Africa, Tunisia and Egypt had the most important number of Pubmed-indexed publications as compared to Morocco followed by Algeria that had the lowest number. As expected, regional high income countries such as France and Spain have significantly contributed to OC research with 3004 and 1577 papers respectively. In the socio-economic analysis as shown in Table 4, Morocco has published 0.09 articles per newly diagnosed OC case based on the incidence data of 2018. Moreover, 0.37 articles were published per million inhabitants and 0.004 OC-related articles per GDP per capita in US-$. Regarding gendermetrics, among the 106 contributing authors found in this entire cluster, 39 only were female researchers. Most of the authors were males (n = 67) with a male to female ratio close to 2. Notably, 8 of the 39 found authors were the first authors who represent the leading researchers of the 13 found items. However, only two female authors were in the last position as principal supervisors.

**Table 4:** Socioeconomic analysis of included articles (core cluster).

Article count	Incidence in 2018	Crude rate in 2018	Number of inhabitants	Gross domestic product (GDP) per capita	Article/new cases	Number of articles published per million inhabitant	Article/GDP per capita
13^†^	139.3	150.6	35, 581, 294^#^	$3,036^#^	0.09	0.37	0.004

^†^ One article from Algeria was excluded. ^#^ 2017 World Bank data (current US$): details can be found at: https://data.worldbank.org/indicator/NY.GDP.PCAP.CD?locations=MA. Abbreviations: GDP: gross domestic product.

### 3.2 Complementary cluster: case reports, case series, and editorials

Table 1 in Supplemental material 1 summarizes the main characteristics of case reports, case series, and editorials. Notably, a first look showed an important number of case reports on rare ovarian tumors which were the most dominant (86%); followed by editorials (9%) and case series (5%) (Figure 2A in Supplemental material 1). These publications were mostly published in French language (70%) as compared to original and review articles (100% in English) (Figure 2B in Supplemental material 1). H Boufettal was the author with the highest number of publications (n=8) in this area. Approximately, 60% of these outputs were covered by Pubmed database and 50% published using the open access model (Figure 2C and D in Supplemental material 1). Historically, the first found case report was published in 2001 by Regragui et al. in “Maroc Médical” and has explored the relationship between appendiceal mucocele, mucinous ovarian tumors and pseudomyxoma peritonei (Regragui et al., 2001). Later, a marked distinctive acceleration of publications was noticed encompassing reports on rare tumors such as granulosa cell tumors, ovarian teratomas, ovarian lymphomas, Demons-Meigs' syndrome and other atypical histological types and anatomic locations.

## 4 Discussion

The role of bibliometrics in evidence-based policy and care delivery is increasingly recognized by health authorities. Knowledge generation in oncology is an important step in the processes of care in every country. This can have a significant impact on patients’ outcomes by guiding and supporting governmental strategies for cancer control. Several methods and indicators are currently used to quantitatively and qualitatively evaluate the scientific literature in specific fields (Joshi, 2014, Ellegaard and Wallin, 2015). Here, some of them were used in our study to make the national research results actionable for elaborating effective future health initiatives for OC.

Our results indicated that between January 2007 and December 2018, Moroccan authors published only two papers that included OC patients. The vast majority of publications were case reports on rare ovarian tumors. The only landmark study that investigated OC in Morocco was conducted by Amrani M et al. in 2014 and has reported the value of immunohistochemical evaluation of various biomarkers on tissue microarray technique in Moroccan patients with benign, borderline and invasive epithelial ovarian tumors (Amrani et al., 2014). Publishing in peer-reviewed Pubmed listed journals is the most widely accepted criterion to measure the scientific outputs and their relevance in medical research. Fortunately, the largest part of the included publications in the two clusters were covered by this database and thus, increasing their international visibility. However, the ultimate goal of publications in oncology is to impact clinical practice through patient-centered outcomes research. This is called “patient impact factor”, which is not achieved yet for our local setting. The number of publications on the genetics of breast and OCs has increased relatively in recent years. This may be explained by the fact that efforts were invested to implement the genetic counseling; particularly with the arrival of genetic profiling techniques such as next-generation sequencing in our country (Belhassan et al., 2016), in addition to the improvements seen in all aspects of public health.

Some of the Moroccan publications on OC were published in several prestigious medical journals with a relatively high impact factor such as *Gynecologic Oncology*, *Cellular Oncology*, *Critical Reviews in Clinical Laboratory Sciences*, and *European Journal of Medicinal Chemistry*. This metric as defined by the annual Journal Citation Reports is still widely used to measure the scientific impact of academic journals, and therefore the published articles, despite several critics for its misuse (Katritsis, 2019). It is a relatively objective approach to quantify and qualify research outputs. The H-index, which is associated with the number of citations, of authors in our study ranged from 2 to 10 only. These low values may be linked to the Matthew effect (Merton, 1968). In fact, it is well known that reputed scientists will be cited more than little-known authors, which is the case of our findings.

International scientific collaboration is defined as a partnership between two or more scientists from two different countries, to complete research tasks with reciprocally shared goals. In our study, regional collaboration with countries from the Mediterranean region was noticed particularly with France. This may be associated with geographical proximity and other factors such as political and economical strategies (Katz, 1994, Chen et al., 2019). When compared to some North African countries, Moroccan and Algerian OC outputs were the lowest. Indeed, Egypt and Tunisia are still leading medical research and especially this field in this African region and have several Pubmed indexed journals intended to publish their national scientific production (Zemni et al., 2018, El Rassi et al., 2018). To date, no Moroccan journal is covered by the Pubmed database. This makes publishing national research in indexed journals difficult. Previously, “Maroc Médical/al-Maghrib al-ṭibbī” journal was the only national journal indexed on Pubmed between 1945 and 1986 and removed later. Therefore, there is an unmet need to develop national medical journals with international standards. When examining Moroccan research productivity standardized by the population size, the number of articles published per million of inhabitants was lower than 1. Similarly, this was also noticed for the number of articles per new cases and per GDP (≈0). Based on the previous density equalizing mapping of the global research architecture on OC worldwide (Brüggmann et al., 2017), our findings seem to be in concordance with the fact that research in this field is concentrated in high-income nations with less involvement of African countries. Notably, Moroccan women's contribution in OC research was less represented. Yet, male’s involvement in the list of authors was remarkably observed. This is in line with the widely recognized issue of gender inequality in science (Chowdhary et al., 2020, Mitchell et al., 2019, Zhou et al., 2018). Importantly, a promising finding of our report is the fact that most of the 13 found articles were published by female scientists as first leading authors despite their under-representation in the list of authors. However, there was a noticeable gap in the number of female contributors as last supervising authors with only two articles in which the last position was given to a female scientist.

Globally, Moroccan researchers produced a very low number of research publications on OC. No article was found regarding the basic epidemiology, clinical and pathological features and survival outcomes of OC. This may be explained by the national prioritization of research on other topics such as breast cancer. In addition, the limited governmental funding and research grants, the lack of health research strategies, as well as the poorly trained workforce in clinical research methods are other reasons. In Morocco, the management of OC involves multidisciplinary teams composed of gynecologists or well trained general surgical oncologists (such as in our center) that perform surgical staging and debulking. Chemotherapy and follow up are ensured by medical oncologists that are the cornerstone of OC treatment in our setting. Radiologists working in public and university hospitals are not well trained to have expertise in oncology and they rarely use the RECIST criteria when evaluating response to chemotherapy. Unfortunately, “Gynecologic Oncology” is not recognized yet as a sub-speciality, which may affect the training of clinicians with expertise in OC management, and therefore enhancing research in this field. Another issue that may halt the publications of national research in international journals is the language. In fact, teaching science courses in Arabic at high school and in French at the university is an important concern in Morocco (Medina, 2015) that is still debated. This is a major barrier with a significant negative impact for clinical researchers as most medical journals publish in English only. A switch to English in medical schools may therefore improve the language background of junior clinicians and facilitate their medical writing skills. In addition, the absence of special research training strategies for clinicians in terms of clinical research methodology may also negatively affect productivity. This is a well-known negative predictor of poor clinical knowledge (Dyrbye et al., 2007). Importantly, enhancing research competencies in the clinical fields is achievable through medical education (Dekker, 2013). Therefore, engaging medical students earlier in targeted programs is an encouraging approach toward research (Naing et al., 2015, Riley et al., 2013). Notably, the previous experience with the implementation of the combined MB/PhD or MD/PhD programs in medical schools in the United Kingdom and France seems to be promising (Hamid et al., 2018, Scherlinger et al., 2018, Lamour et al., 2018, Barnett-Vanes et al., 2015). This is urgently needed in Morocco to improve the research background of healthcare professionals. Additionally, the establishment of research networks and working groups such as *The Ovarian Cancer Association Consortium* (OCAC) founded in 2005 is a nice example for boosting research on OC globally (http://ocac.ccge.medschl.cam.ac.uk/). This project has allowed a multidisciplinary and international collaboration between oncologists and published more than 150 papers until today. Thus, creating working groups and scientific societies should be implemented in developing countries such as Morocco.

To the best of our knowledge, this study is the first bibliometric analysis focusing on OC trends in Morocco. The data downloaded from the available sources, including Pubmed, covered the vast majority of articles in the field of OC research. We also included the Francophone literature to limit any language biases and to provide a broader range of coverage. However, since medical theses, conference proceedings, patents, and books have not been included in document screening; our data may not represent the whole picture of this topic in Morocco. Also, because the number of publications found was small, we used manual data extraction which may increase the risk of human error in our report. Moreover, bibliometric indicators have several limitations (reviewed elsewhere: (Belter, 2015) and therefore, caution should be taken during their interpretation. As such, the peer-review of the found items cannot be easily assessed as most journals don’t share the related reports publically. This is an important qualitative parameter particularly with the recent emergence of prolific predatory journals. Finally, the VOSviewer software for bibliometric analysis was not used given the small number of studies found in our screening. Promisingly, the findings of this first bibliometric study on OC in Morocco are expected to provide useful information for those who will be performing clinical and translational studies in the near future and also for health authorities.

## 5 Conclusions

This bibliometric analysis demonstrated that there are limited research contributions on OC in Morocco. This provided a preliminary description of the scientific productivity on this topic, which was largely dominated by case reports and case series on rare ovarian tumors. Scientific research publications on OC in Morocco are lacking particularly in the area of medical oncology. Promisingly, a clinical and translational project (OVANORDEST 1 and OVANORDEST 2 studies) to develop research on OC in Morocco was started by our team in 2019 and it is expected to be finalized in the next few years. This will certainly boost research outputs in this area in the future. We have also created the *Cancer Biomarkers Working Group* to increase national and international collaboration on this topic. Furthermore, a project to launch a Moroccan journal with an international publisher is being discussed. A re-evaluation of the published literature on OC research in Morocco is being programmed for the next few years.

## 6 Ethics approval and consent to participate

Not applicable.

## 7 Consent for publication

Not applicable.

## 8 Availability of data and materials

All data described in this article can be retrieved from bibliographic databases using keywords listed in our methods section. The Excel file can be shared upon request from the corresponding author.

## 9 Funding

Open access fees were provided by a research grant from the ‘’Cancer Research Institute IRC’’, Kingdom of Morocco (www.irc.ma).

## 10 Authors' contributions

KE conducted the bibliometric study and wrote the manuscript under the supervision of Profs. OA and SA. All authors read the final version of the manuscript.
