# Supplementary material for: Tracing ovarian cancer research in Morocco: A bibliometric analysis
Source: Gynecol Oncol Rep. 2021 May 7;37:100777. doi: 10.1016/j.gore.2021.100777 (PMC8192560; doi:10.1016/j.gore.2021.100777)
Supplement: Supplementary data 1 [file mmc1.pdf]

Supplemental Material 2 (Turnitin Report of Plagiarism/El Bairi et al.)

by Khalid El Bairi

Submission date: 19-Apr-2021 01:15AM (UTC+0100)
Submission ID: 104409528
File name: For_turnitn.docx (64.44K)
Word count: 5201
Character count: 31229

Tracing Ovarian Cancer Research in Morocco: A Bibliometric Analysis

Abstract:

Background: The burden of ovarian cancer (OC) in low-income countries continues to increase annually. This gynecological cancer, known for its poor survival outcomes, has not attracted much interest in medical research as compared to other women's malignancies such as breast cancer. This bibliometric study was conducted to better depict the global map and the future directions of scientific productivity in the area of OC research in Morocco.

Methods: Publication trends on OC were retrospectively analyzed using a number of bibliometric parameters based on the Pubmed database and other resources.

Results: During the time period (1900-2018), a total number of 74 publications responding to the inclusion criteria were found and incorporated in the bibliometric analysis. This was dominated by case reports and case series on rare ovarian tumors (n=60). In the core cluster, only 10 original studies and 3 reviews on OC were published by Moroccan researchers. After full-text appraisal for study population, only two clinical original articles included OC patients. The other clinical studies included breast cancer patients only or were suggestive of inherited OC. In addition, 3 preclinical *in vitro* studies were found during the literature search. The majority of these publications were covered by Pubmed and Web of Science core collection and all published in English language. The H-index of top 10 Moroccan scientists in this area didn't exceed 10. Importantly, research and review articles were frequently published in influential journals. However, the number of publications as compared to other African countries was very low. Moreover, a similar trend in terms of article per each newly diagnosed OC case, GDP per capita and per million was also noticed. For gender distribution, female scientists were first authors in the majority of these papers but less represented as leading last authors. In the complementary cluster of other article types on rare ovarian tumors, 70% of the items were published in French and approximately 60% were indexed on Pubmed. During the last five years, a marked acceleration of publishing this research category with little impact in the evidence-based practice was noticed.

Conclusions: This research area in gynecologic oncology seems to be neglected and needs to be prioritized in future research projects in Morocco particularly given the aggressive behavior of this women's cancer and the few available therapeutic options. There is an unmet need for studies on OC in all fields particularly epidemiology, clinic-pathological characteristics, and survival outcomes.

Keywords: Ovarian cancer, cancer research, bibliometric, scientometric, Morocco

Introduction

Historically, "*Bibliometrics*" was first introduced into the literature by the Belgian librarian Paul Otlet in his book "*Traité de Documentation*" in 1934 [1]. The author defined this new term as "*the measurement of all aspects related to the publication and reading of books and documents*" [1,2]. Since then, an important number of bibliometric reports to analyze the structure of the published literature in various areas in science were published, particularly in medicine with more than 12,400 articles found on Pubmed (as of 11-07-2020) [3,4]. Bibliometrics plays a significant role in the quantitative and qualitative assessment of research landscapes of particular fields [5,6]. This may considerably impact research projects, guide the design of future studies, and boost the national contribution in scientific productivity to achieve superior international visibility. Moreover, bibliometric investigations have also a powerful role in governmental policies and strategies to improve and support decision-making, disease control, and patients' care [7,8].

Ovarian cancer (OC) is still a leading cause of high rates of mortality from gynecological cancers [9]. OC is the 7th most common cancer and the 8th in terms of mortality among women worldwide [9-11]. According to the latest updates of the GLOBOCAN database (available at: https://gco.iarc.fr/), OC in Morocco is the third gynecological cancer in terms of incidence and is ranked 15 with 1222 new cases for both sexes in 2020 for all cancer sites. OC has a 5-years prevalence of 15.81 per 100,000 and is also the third gynecological cancer in terms of mortality. Despite recent advances in therapy, OC has 5-year relative survival below 45% [9]. This is mainly due to the diagnosis in advanced stages and resistance to the standard platinum-based chemotherapy. The marked poor prognostic outcomes observed in

this women's cancer have raised awareness toward advancing clinical and translational research to uncover the mechanisms of this aggressive disease, develop early detection strategies, and find additional therapeutics beyond platinum-based combinations. Promisingly, the published research related to OC is continuously increasing [12]. However, this trend is concentrated in high-income countries as compared to lower-resources nations [12]. Previously, only two bibliometric studies have provided a mapping of the research architecture of OC research in Turkey [13] and globally [12]. Unfortunately, almost no visible role in the productivity of African OC publications in the scientific community has been noticed despite the high burden of this disease in their population [12].

Our report aims to provide a global overview of OC research in Morocco. The study period was fixed between 2018 and other previous years before we started our projects to develop research on OC in our setting. Hypothetically, this is anticipated to examine the need for additional research on specific topics in this area. To the best of our knowledge, this is the third bibliometric report on OC research worldwide and the first to be conducted in Morocco.

Methods

Search strategy

We used abstracting/indexing engines and full-text databases to find published articles on OC by Moroccan scientists retrospectively. Additionally, other sources including cross-referencing and Google Scholar were checked to find more publications. The search strategy is described as follows: advanced search on Pubmed/Medline (National Center for Biotechnology Information), which covers most of the medical journals, and Scopus (Elsevier®) using the following combinations of keywords: "ovarian carcinoma", OR "cancer of the ovary", OR "ovarian malignancy", OR "ovarian tumor", OR "ovarian neoplasm" AND "Morocco". The MeSH database was also searched: ("ovarian Neoplasms"[Mesh]) AND "Morocco"[Mesh]. Moreover, additional searches based on cross-referencing, SpringerLink (Springer Nature®), and Google Scholar using the same previous keywords were screened and provided other papers not covered by Pubmed. To limit language bias, EM-Consulte (Elsevier

Masson®) and ScienceDirect (Elsevier®) were selected to cover the Francophone literature. Journal Citation Reports™ 2020 (Clarivate Analytics) was used to find updated journal impact factors. A 1900 (01-01) to 2018 (30-12) analysis of studies that focused on ovarian malignancies was used to find relevant articles. The period selection was chosen to fit the start of our project to develop research on OC in 2018. Selected articles were preliminary checked for eligibility based on their titles and abstracts, and then fully verified for OC patients' inclusion in their study population. Only peer-reviewed and published papers during the period 1900-2018 were selected (**Figure 1**). Ongoing studies and pre-prints from ClinicalTrials.gov, ResearchSquare, and medRxiv were excluded. In an attempt for comparison with other cancer types and research outputs in other countries, only Pubmed search was used. Bibliographic searching based on these criteria was run independently twice.

The GLOBOCAN official website (Cancer Today-IARC: available at: https://gco.iarc.fr/today/online-analysis-map) was used to collect the absolute numbers of OC age-standardized incidence, and the crude rate which is calculated by dividing the number of new cases for a specific cancer observed during a given time period by the corresponding number of person years in the population at risk (usually expressed as an annual rate per 100,000 persons at risk) [14]. We then calculated the ratio of country-specific articles per each new OC case in Morocco as previously described [12] and per million inhabitants. Moreover, a socioeconomic quantification of country-specific contributions concerns the economic resources which were investigated based on the gross domestic product (GDP) per capita, and population size using the databases of The World Bank (available at: https://data.worldbank.org/indicator/NY.GDP.PCAP.CD?locations=MA). Finally, we defined international collaboration when at least one author with a foreign affiliation had contributed to the study.

Data analysis and categorization

Data related to: author/year, article title, article type, article language, journal, open access or subscription model, Pubmed indexing, Web of Science (WoS) indexing**,** research field, total number of authors, total number of female authors, male-female ratio, funding and international collaboration (for reviews and original articles only),

journal impact factors according to the latest version of Journal Citation Reports 2020, and first authors' H-index according to Scopus database (as of 15-07-2020), were extracted and summarized in 4 different tables. Firstly, data were extracted manually and analyzed using Microsoft Office Excel 2007 (Microsoft, Redmond, WA, USA) for basic statistics. For a better qualitative assessment, our findings were categorized into two clusters of papers including a cluster of reviews and original articles and an additional cluster of case reports, case series, and editorials for rare ovarian malignancies. This was conducted for a better assessment of article types. Ethical committee approval was not required as the study design is based on available published research. Publications authors were not contacted for further information regarding their published studies.

Results

Core cluster: research and review articles

During the study period, 14 articles encompassing 10 original articles and 4 reviews were included in the selection/verification process (**Table 1**, **Figure 2**). Eight of them were published using the open access model. Only one publication [20] was not indexed on Pubmed. For WoS, two papers published in *Breast Disease* and *Biomedical Engineering Research* [20,25] were not found on this highly selective database. All of these articles were published in English language and were mostly in the clinical area (11/14) and half of these publications declared receiving funding from national and international organizations. Regarding international collaboration, 9 of the 14 publications included at least one author from a foreign affiliation, mainly from the European Union (France and Italy). **Table 2** shows the evolution of publications by research field and funding. During the period 2000-2010, no article in the clinical area was found. Thereafter, 11 publications were found including 7 original articles and 3 reviews in addition to another review from an Algerian team that described the *BRCA* mutational status in hereditary breast and OCs in the Maghrebian countries by Cherbal et al. [25]. Notably, of these 7 original studies, only two reports had OC patients in their study population [20,24]. Similarly, fundamental research papers were rarely observed. Three *in vitro* studies that investigated the

pharmacological activities of natural and synthetic compounds on OC cells were found during this period [12,26,28].

Regarding the H-index (**Table 3**), which is an important parameter to measure the eminence of researchers, the top ten Moroccan researcher's H-index ranged from 2 (Jouali F and Sekkate S, geneticist and medical oncologist, respectively) to 10 (Amrani M, pathologist). For journal impact factors according to the latest Journal Citation Reports (Clarivate Analytics®), there was a trend in publishing in prestigious journals from respected academic publishers. The highest impact factor was noticed for a preclinical study by Abbassi N et al. (2012) (5.572/European Journal of Medicinal Chemistry) [26], followed by El Bairi K et al. (2017) (5.304/Cellular Oncology) [16], El Bairi K et al. (2017) (4.677/Critical Reviews in Clinical Laboratory Sciences) [17], and Tazzite A et al. (2012) (4.623/Gynecologic Oncology) [24] (details can be found in **Table 3**).

Because we didn't find any recent bibliometric study on this topic from North African countries, we used Pubmed to map the global landscape of research on OC in general. This pre-screening strategy has several limitations such as the significant overlap with other ovarian tumors particularly rare diseases. From this viewpoint, we aimed to compare Moroccan contributions with other regional countries such as Egypt, Spain, France, Algeria, Tunisia, and some other African countries as shown in **Figure 1 in Supplemental 1**. In North Africa, Tunisia and Egypt had the most important number of Pubmed-indexed publications as compared to Morocco followed by Algeria that had the lowest number. As expected, regional high income countries such as France and Spain have significantly contributed to OC research with 3004 and 1577 papers respectively. In the socio-economic analysis as shown in **Table 4**, Morocco has published 0.09 articles per newly diagnosed OC case based on the incidence data of 2018. Moreover, 0.37 articles were published per million inhabitants and 0.004 OC-related articles per GDP per capita in US-$. Regarding gendermetrics, among the 106 contributing authors found in this entire cluster, 39 only were female researchers. Most of the authors were males (n=67) with a male to female ratio close to 2. Notably, 8 of the 39 found authors were the first authors who represent the leading researchers of the 13 found items. However, only two female authors were in the last position as principal supervisors.

Complementary cluster: case reports, case series, and editorials

Table 1 in Supplemental material 1 summarizes the main characteristics of case reports, case series, and editorials. Notably, a first look showed an important number of case reports on rare ovarian tumors which were the most dominant (86%); followed by editorials (9%) and case series (5%) (**Figure 2A in Supplemental material 1**). These publications were mostly published in French language (70%) as compared to original and review articles (100% in English) (**Figure 2B in Supplemental material 1**). H Boufettal was the author with the highest number of publications (n=8) in this area. Approximately, 60% of these outputs were covered by Pubmed database and 50% published using the open access model (**Figure 2C and D in Supplemental material 1**). Historically, the first found case report was published in 2001 by Regragui et al. in "Maroc Médical" and has explored the relationship between appendiceal mucocele, mucinous ovarian tumors and pseudomyxoma peritonei [29]. Later, a marked distinctive acceleration of publications was noticed encompassing reports on rare tumors such as granulosa cell tumors, ovarian teratomas, ovarian lymphomas, Demons-Meigs' syndrome and other atypical histological types and anatomic locations.

Discussion

The role of bibliometrics in evidence-based policy and care delivery is increasingly recognized by health authorities. Knowledge generation in oncology is an important step in the processes of care in every country. This can have a significant impact on patients' outcomes by guiding and supporting governmental strategies for cancer control. Several methods and indicators are currently used to quantitatively and qualitatively evaluate the scientific literature in specific fields [30,31]. Here, some of them were used in our study to make the national research results actionable for elaborating effective future health initiatives for OC.

Our results indicated that between January 2007 and December 2018, Moroccan authors published only two papers that included OC patients. The vast majority of publications were case reports on rare ovarian tumors. The only landmark study that

investigated OC in Morocco was conducted by Amrani M et al. in 2014 and has reported the value of immunohistochemical evaluation of various biomarkers on tissue microarray technique in Moroccan patients with benign, borderline and invasive epithelial ovarian tumors [20]. Publishing in peer-reviewed Pubmed listed journals is the most widely accepted criterion to measure the scientific outputs and their relevance in medical research. Fortunately, the largest part of the included publications in the two clusters were covered by this database and thus, increasing their international visibility. However, the ultimate goal of publications in oncology is to impact clinical practice through patient-centered outcomes research. This is called “patient impact factor”, which is not achieved yet for our local setting. The number of publications on the genetics of breast and OCs has increased relatively in recent years. This may be explained by the fact that efforts were invested to implement the genetic counseling; particularly with the arrival of genetic profiling techniques such as next-generation sequencing in our country [32], in addition to the improvements seen in all aspects of public health.

Some of the Moroccan publications on OC were published in several prestigious medical journals with a relatively high impact factor such as *Gynecologic Oncology*, *Cellular Oncology*, *Critical Reviews in Clinical Laboratory Sciences*, and *European Journal of Medicinal Chemistry*. This metric as defined by the annual Journal Citation Reports is still widely used to measure the scientific impact of academic journals, and therefore the published articles, despite several critics for its misuse [33]. It is a relatively objective approach to quantify and qualify research outputs. The H-index, which is associated with the number of citations, of authors in our study ranged from 2 to 10 only. These low values may be linked to the Matthew effect [34]. In fact, it is well known that reputed scientists will be cited more than little-known authors, which is the case of our findings.

International scientific collaboration is defined as a partnership between two or more scientists from two different countries, to complete research tasks with reciprocally shared goals. In our study, regional collaboration with countries from the Mediterranean region was noticed particularly with France. This may be associated with geographical proximity and other factors such as political and economical strategies [35,36]. When compared to some North African countries, Moroccan and Algerian OC outputs were the lowest. Indeed, Egypt and Tunisia are still leading

medical research and especially this field in this African region and have several Pubmed indexed journals intended to publish their national scientific production [37,38]. To date, no Moroccan journal is covered by the Pubmed database. This makes publishing national research in indexed journals difficult. Previously, "Maroc Médical/al-Maghrib al-ṭibbī" journal was the only national journal indexed on Pubmed between 1945 and 1986 and removed later. Therefore, there is an unmet need to develop national medical journals with international standards. When examining Moroccan research productivity standardized by the population size, the number of articles published per million of inhabitants was lower than 1. Similarly, this was also noticed for the number of articles per new cases and per GDP (≈0). Based on the previous density equalizing mapping of the global research architecture on OC worldwide [12], our findings seem to be in concordance with the fact that research in this field is concentrated in high-income nations with less involvement of African countries. Notably, Moroccan women's contribution in OC research was less represented. Yet, male's involvement in the list of authors was remarkably observed. This is in line with the widely recognized issue of gender inequality in science [39-41]. Importantly, a promising finding of our report is the fact that most of the 13 found articles were published by female scientists as first leading authors despite their under-representation in the list of authors. However, there was a noticeable gap in the number of female contributors as last supervising authors with only two articles in which the last position was given to a female scientist.

Globally, Moroccan researchers produced a very low number of research publications on OC. No article was found regarding the basic epidemiology, clinical and pathological features and survival outcomes of OC. This may be explained by the national prioritization of research on other topics such as breast cancer. In addition, the limited governmental funding and research grants, the lack of health research strategies, as well as the poorly trained workforce in clinical research methods are other reasons. In Morocco, the management of OC involves multidisciplinary teams composed of gynecologists or well trained general surgical oncologists (such as in our center) that perform surgical staging and debulking. Chemotherapy and follow up are ensured by medical oncologists that are the cornerstone of OC treatment in our setting. Radiologists working in public and university hospitals are not well trained to have expertise in oncology and they rarely

use the RECIST criteria when evaluating response to chemotherapy. Unfortunately, “Gynecologic Oncology” is not recognized yet as a sub-speciality, which may affect the training of clinicians with expertise in OC management, and therefore enhancing research in this field. Another issue that may halt the publications of national research in international journals is the language. In fact, teaching science courses in Arabic at high school and in French at the university is an important concern in Morocco [42] that is still debated. This is a major barrier with a significant negative impact for clinical researchers as most medical journals publish in English only. A switch to English in medical schools may therefore improve the language background of junior clinicians and facilitate their medical writing skills. In addition, the absence of special research training strategies for clinicians in terms of clinical research methodology may also negatively affect productivity. This is a well-known negative predictor of poor clinical knowledge [43]. Importantly, enhancing research competencies in the clinical fields is achievable through medical education [44]. Therefore, engaging medical students earlier in targeted programs is an encouraging approach toward research [45,46]. Notably, the previous experience with the implementation of the combined MB/PhD or MD/PhD programs in medical schools in the United Kingdom and France seems to be promising [47-50]. This is urgently needed in Morocco to improve the research background of healthcare professionals. Additionally, the establishment of research networks and working groups such as *The Ovarian Cancer Association Consortium* (OCAC) founded in 2005 is a nice example for boosting research on OC globally (http://ocac.ccge.medschl.cam.ac.uk/). This project has allowed a multidisciplinary and international collaboration between oncologists and published more than 150 papers until today. Thus, creating working groups and scientific societies should be implemented in developing countries such as Morocco.

To the best of our knowledge, this study is the first bibliometric analysis focusing on OC trends in Morocco. The data downloaded from the available sources, including Pubmed, covered the vast majority of articles in the field of OC research. We also included the Francophone literature to limit any language biases and to provide a broader range of coverage. However, since medical theses, conference proceedings, patents, and books have not been included in document screening; our data may not represent the whole picture of this topic in Morocco. Also, because the number of

publications found was small, we used manual data extraction which may increase the risk of human error in our report. Moreover, bibliometric indicators have several limitations (reviewed elsewhere: [6]) and therefore, caution should be taken during their interpretation. As such, the peer-review of the found items cannot be easily assessed as most journals don't share the related reports publically. This is an important qualitative parameter particularly with the recent emergence of prolific predatory journals. Finally, the VOSviewer software for bibliometric analysis was not used given the small number of studies found in our screening. Promisingly, the findings of this first bibliometric study on OC in Morocco are expected to provide useful information for those who will be performing clinical and translational studies in the near future and also for health authorities.

Conclusions

This bibliometric analysis demonstrated that there are limited research contributions on OC in Morocco. This provided a preliminary description of the scientific productivity on this topic, which was largely dominated by case reports and case series on rare ovarian tumors. Scientific research publications on OC in Morocco are lacking particularly in the area of medical oncology. Promisingly, a clinical and translational project (OVANORDEST 1 and OVANORDEST 2 studies) to develop research on OC in Morocco was started by our team in 2019 and it is expected to be finalized in the next few years. This will certainly boost research outputs in this area in the future. We have also created the *Cancer Biomarkers Working Group* to increase national and international collaboration on this topic. Furthermore, a project to launch a Moroccan journal with an international publisher is being discussed. A re-evaluation of the published literature on OC research in Morocco is being programmed for the next few years.

List of abbreviations

BRCA: Breast cancer susceptibility gene, GDP: gross domestic product, IARC: International Agency for Research on Cancer, OC: ovarian cancer; OCAC: The Ovarian Cancer Association Consortium, US: United States, WoS: Web of Science.

References

1. Rousseau R. Library science: Forgotten founder of bibliometrics. Nature. 2014;510(7504):218. doi:10.1038/510218e.

2. Otlet, P. Traité de documentation. Le livre sur le livre, 1934. Palais Mondial, Bruxelles.

3. Kokol P, Blažun Vošner H, Završnik J. Application of bibliometrics in medicine: a historical bibliometrics analysis. Health Info Libr J. 2020;10.1111/hir.12295. doi:10.1111/hir.12295

4. https://pubmed.ncbi.nlm.nih.gov/?term=bibliometric&sort=date&size=50 (accessed 11/07/2020).

5. Haustein S, Larivière V. The use of bibliometrics for assessing research: possibilities, limitations and adverse effects. 2015. In Incentives and performance, Springer, 121–139.

6. Belter CW. Bibliometric indicators: opportunities and limits. J Med Libr Assoc. 2015;103(4):219-221. doi:10.3163/1536-5050.103.4.014.

7. Ismail S, Nason E, Marjanovic S, Grant J. Bibliometrics as a Tool for Supporting Prospective R&D Decision-Making in the Health Sciences: Strengths, Weaknesses and Options for Future Development. Rand Health Q. 2012;1(4):11.

8. Thompson DF, Walker CK. A descriptive and historical review of bibliometrics with applications to medical sciences. Pharmacotherapy. 2015;35(6):551-559. doi:10.1002/phar.1586.

9. Webb PM, Jordan SJ. Epidemiology of epithelial ovarian cancer. Best Pract Res Clin Obstet Gynaecol. 2017;41:3-14. doi:10.1016/j.bpobgyn.2016.08.006.

10. Coburn SB, Bray F, Sherman ME, Trabert B. International patterns and trends in ovarian cancer incidence, overall and by histologic subtype. Int J Cancer. 2017;140(11):2451-2460. doi:10.1002/ijc.30676.

11. Momenimovahed Z, Tiznobaik A, Taheri S, Salehiniya H. Ovarian cancer in the world: epidemiology and risk factors. Int J Womens Health. 2019;11:287-299. doi:10.2147/IJWH.S197604.

12. Brüggmann D, Pulch K, Klingelhöfer D, Pearce CL, Groneberg DA. Ovarian cancer: density equalizing mapping of the global research architecture. Int J Health Geogr. 2017;16(1):3. doi:10.1186/s12942-016-0076-2.

13. Guler T, Yayci E, Atacag T, Cetin A. An analysis of Turkey's scientific contribution in ovarian cancer research. Eur J Gynaecol Oncol. 2013;34(2):175-178.

14. https://www-dep.iarc.fr/whodb/glossary.htm (accessed 23/07/2020)

15. Laarabi FZ, Ratbi I, Elalaoui SC, et al. High frequency of the recurrent c.1310_1313delAAGA BRCA2 mutation in the North-East of Morocco and implication for hereditary breast-ovarian cancer prevention and control. BMC Res Notes. 2017;10(1):188. Published 2017 Jun 2. doi:10.1186/s13104-017-2511-2.

16. El Bairi K, Kandhro AH, Gouri A, et al. Emerging diagnostic, prognostic and therapeutic biomarkers for ovarian cancer. Cell Oncol (Dordr). 2017;40(2):105-118. doi:10.1007/s13402-016-0309-1.

17. El Bairi K, Amrani M, Kandhro AH, Afqir S. Prediction of therapy response in ovarian cancer: Where are we now?. Crit Rev Clin Lab Sci. 2017;54(4):233-266. doi:10.1080/10408363.2017.1313190.

18. Jouali F, Laarabi FZ, Marchoudi N, et al. First application of next-generation sequencing in Moroccan breast/ovarian cancer families and report of a novel frameshift mutation of the BRCA1 gene. Oncol Lett. 2016;12(2):1192-1196. doi:10.3892/ol.2016.4739.

19. Laraqui A, Uhrhammer N, Rhaffouli HE, et al. BRCA genetic screening in Middle Eastern and North African: mutational spectrum and founder BRCA1 mutation (c.798_799delTT) in North African. Dis Markers. 2015;2015:194293. doi:10.1155/2015/194293.

20. Amrani M, Memeo L, Kadiri H, Charhi H, Belabbas MA, Mansukhani MM. Immunohistochemical Analysis of WT1, EGFR, E-cadherin, beta-catenin and p53 in 43 Moroccan Epithelial Ovarian Tumours. Biomedical Engineering Research. 2014;3:11–17.

21. Abbassi N, Rakib el M, Chicha H, et al. Synthesis and antitumor activity of some substituted indazole derivatives. Arch Pharm (Weinheim). 2014;347(6):423-431. doi:10.1002/ardp.201300390.

22. Sekkate S, Kairouani M, Serji B, et al. Ovarian granulosa cell tumors: a retrospective study of 27 cases and a review of the literature. World J Surg Oncol. 2013;11:142. Published 2013 Jun 18. doi:10.1186/1477-7819-11-142.

23. Laraqui A, Uhrhammer N, Lahlou-Amine I, et al. Mutation screening of the BRCA1 gene in early onset and familial breast/ovarian cancer in Moroccan population. Int J Med Sci. 2013;10(1):60-67. doi:10.7150/ijms.5014.

24. Tazzite A, Jouhadi H, Nadifi S, et al. BRCA1 and BRCA2 germline mutations in Moroccan breast/ovarian cancer families: novel mutations and unclassified variants. Gynecol Oncol. 2012;125(3):687-692. doi:10.1016/j.ygyno.2012.03.007.

25. Cherbal F, Bakour R, Adane S, Boualga K. BRCA1 and BRCA2 germline mutation spectrum in hereditary breast/ovarian cancer families from Maghrebian countries. Breast Dis. 2012;34(1):1-8. doi:10.3233/BD-130348.

26. Abbassi N, Chicha H, Rakib el M, et al. Synthesis, antiproliferative and apoptotic activities of N-(6(4)-indazolyl)-benzenesulfonamide derivatives as potential anticancer agents. Eur J Med Chem. 2012;57:240-249. doi:10.1016/j.ejmech.2012.09.013.

27. Laarabi FZ, Jaouad IC, Ouldim K, et al. Genetic testing and first presymptomatic diagnosis in Moroccan families at high risk for breast/ovarian cancer. Oncol Lett. 2011;2(2):389-393. doi:10.3892/ol.2011.248.

28. Ait M'barek L, Ait Mouse H, Jaâfari A, et al. Cytotoxic effect of essential oil of thyme (Thymus broussonettii) on the IGR-OV1 tumor cells resistant to chemotherapy. Braz J Med Biol Res. 2007;40(11):1537-1544. doi:10.1590/s0100-879x2007001100014.

29. Regragui A, Amrani M, Laraqui L, et al. Relationship between appendiceal mucocele, mucinous ovarian tumours and peritoneal gelatinous disease. Maroc Med. 2001;23(3).

30. Joshi MA. Bibliometric indicators for evaluating the quality of scientifc publications. J Contemp Dent Pract. 2014;15(2):258-262. Published 2014 Mar 1. doi:10.5005/jp-journals-10024-1525.

31. Ellegaard O, Wallin JA. The bibliometric analysis of scholarly production: How great is the impact?. Scientometrics. 2015;105(3):1809-1831. doi:10.1007/s11192-015-1645-z.

32. Belhassan K, Ouldim K, Sefiani AA. Genetics and genomic medicine in Morocco: the present hope can make the future bright. Mol Genet Genomic Med. 2016;4(6):588-598. doi:10.1002/mgg3.255.

33. Katritsis DG. Journal Impact Factor: Widely Used, Misused and Abused. Arrhythm Electrophysiol Rev. 2019;8(3):153-155. doi:10.15420/aer.2019.8.3.FO1.

34. Merton RK. The Matthew effect in science. The reward and communication systems of science are considered. Science. 1968;159(3810):56-63.

35. Katz J. Geographical proximity and scientific collaboration. Scientometrics. 1994;31(1): 31-43.

36. Chen K, Zhang Y, Fu X. International research collaboration: an emerging domain of innovation studies?. Res Policy. 2019; 48;149e168. doi :10.1016/ j.respol.2018.08.005.

37. Zemni I, Safer M, Horrigue I, Ben Abdelaziz A, Hammami S, Ben Abdelaziz A. Bibliometric profile of Tunisian medical publications written in English and indexed in Medline. Tunis Med. 2018;96(7):411-416.

38. El Rassi R, Meho LI, Nahlawi A, Salameh JS, Bazarbachi A, Akl EA. Medical research productivity in the Arab countries: 2007-2016 bibliometric analysis. J Glob Health. 2018;8(2):020411. doi:10.7189/jogh.08.020411.

39. Chowdhary M, Chowdhary A, Royce TJ, et al. Women's Representation in Leadership Positions in Academic Medical Oncology, Radiation Oncology, and Surgical Oncology Programs. JAMA Netw Open. 2020;3(3):e200708. doi:10.1001/jamanetworkopen.2020.0708.

40. Mitchell CA, Roussel MF, Walsh L, Weeraratna AT. Women in cancer research. Nat Rev Cancer. 2019;19(10):547-552. doi:10.1038/s41568-019-0176-y.

41. Zhou CD, Head MG, Marshall DC, et al. A systematic analysis of UK cancer research funding by gender of primary investigator. BMJ Open. 2018;8(4):e018625. doi:10.1136/bmjopen-2017-018625.

42. Medina F. The output of researchers in Morocco compared to some North African countries from 1996 to 2012, and its relationship to governmental major decisions on higher education and scientific research. Scientometrics. 2015;105(1), 367–384. doi :10.1007/s11192-015-1701-8.

43. Dyrbye LN, Thomas MR, Natt N, Rohren CH. Prolonged delays for research training in medical school are associated with poorer subsequent clinical knowledge. J Gen Intern Med. 2007;22(8):1101-1106. doi:10.1007/s11606-007-0200-x.

44. Dekker FW. Achieving research competences through medical education. Perspect Med Educ. 2013;2(4):178-180. doi:10.1007/s40037-013-0084-x.

45. Naing C, Wai VN, Durham J, et al. A Systematic Review and Meta-Analysis of Medical Students' Perspectives on the Engagement in Research. Medicine (Baltimore). 2015;94(28):e1089. doi:10.1097/MD.0000000000001089.

46. Riley SC, Morton J, Ray DC, Swann DG, Davidson DJ. An integrated model for developing research skills in an undergraduate medical curriculum: appraisal of an approach using student selected components. Perspect Med Educ. 2013;2(4):230-247. doi:10.1007/s40037-013-0079-7.

47. Hamid O, Burhan R, Cheng LH. Should UK medical students complete a PhD during their undergraduate studies?. Adv Med Educ Pract. 2018;9:727-728. Published 2018 Sep 28. doi:10.2147/AMEP.S172844.

48. Scherlinger M, Bienvenu TCM, Piffoux M, Séguin P. Les doubles cursus médecine-sciences en France - État des lieux et perspectives [MD-PhD trainings in France: overview and future directions, from the French MD/PharmD-PhD students association « AMPS »]. Med Sci (Paris). 2018;34(5):464-472. doi:10.1051/medsci/20183405021.

49. Lamour V, Bessereau JL, Thalabard JC, et al. Le réseau national des filières médecine-sciences [French network of combined MD-PhD degree programs]. Med Sci (Paris). 2018;34(5):462-463. doi:10.1051/medsci/20183405020.

50. Barnett-Vanes A, Ho G, Cox TM. Clinician-scientist MB/PhD training in the UK: a nationwide survey of medical school policy. BMJ Open. 2015;5(12):e009852. Published 2015 Dec 30. doi:10.1136/bmjopen-2015-009852.

Figure legends

Figure 1. Flow chart of article selection

Figure 2. Evolution of number of original and review articles per year

Supplemental Material 2 (Turnitin Report of Plagiarism/El Bairi et al.)

ORIGINALITY REPORT

29%	24%	26%	15%
SIMILARITY INDEX	INTERNET SOURCES	PUBLICATIONS	STUDENT PAPERS

PRIMARY SOURCES

1	www.dovepress.com<br>Internet Source	2%
2	journals.plos.org<br>Internet Source	1%
3	Solomon O. Rotimi, Oluwakemi A. Rotimi, Bodour Salhia. "A Review of Cancer Genetics and Genomics Studies in Africa", Frontiers in Oncology, 2021<br>Publication	1%
4	Mohammed Mansouri, Touria Derkaoui, Joaira Bakkach, Ali Loudiyi et al. "Screening of BRCA1 and BRCA2 germline mutations in unselected triple‐negative breast cancer patients: A series from north of Morocco", Precision Medical Sciences, 2020<br>Publication	1%
5	www.ijhpm.com<br>Internet Source	1%
6	ij-healthgeographics.biomedcentral.com<br>Internet Source	1%

7 www.medecinesciences.org
Internet Source
1 %

8 Shiqi Zhang, Dongyi Zhao, Wanying Jia, Yuting Wang, Hongyue Liang, Lei Liu, Wuyang Wang, Zhiyi Yu, Feng Guo. "A bibliometric analysis and review of recent researches on TRPM7", Channels, 2020
Publication
1 %

9 Hanae Lrhoul, Naïma El Assaoui, Houcemeddine Turki. "Mapping of water research in Morocco: A scientometric analysis", Materials Today: Proceedings, 2021
Publication
1 %

10 journals.viamedica.pl
Internet Source
1 %

11 applications.emro.who.int
Internet Source
1 %

12 jamanetwork.com
Internet Source
1 %

13 Khalid El Bairi, Rachid Jabi, Dario Trapani, Hanae Boutallaka et al. "Can the microbiota predict response to systemic cancer therapy, surgical outcomes, and survival? The answer is in the gut.", Expert Review of Clinical Pharmacology, 2020
Publication
1 %

14	www.impactjournals.com<br>Internet Source	1 %
15	www.researchsquare.com<br>Internet Source	1 %
16	www.thefreelibrary.com<br>Internet Source	1 %
17	Robin J. Jacobs, Michael Kane. "Predictors of Research Self Efficacy in First-year Osteopathic Medical Students", International Journal of Osteopathic Medicine, 2020<br>Publication	1 %
18	onlinelibrary.wiley.com<br>Internet Source	1 %
19	Assoman Kouakou, Hakima Chicha, El Mostapha Rakib, Ahmed Gamouh, Abdellah Hannioui, Mohammed Chigr, Maurizio Viale. " SnCl /RSH: a versatile catalytic system for the synthesis of 4-alkylsulfanyl-indazole derivatives ", Journal of Sulfur Chemistry, 2014<br>Publication	1 %
20	Adnin Zaman, Micol S. Rothman. "Postmenopausal Hyperandrogenism", Endocrinology and Metabolism Clinics of North America, 2021<br>Publication	<1 %

21 Alsmawal A. Elimam, Mohamed Elmogtba Mouaweia Mohamed Aabdein, Mohamed El-Fatih Moly Eldeen, Hisham N. Altayb et al. "Monoallelic characteristic-bearing heterozygous L1053X in BRCA2 gene among Sudanese women with breast cancer", BMC Medical Genetics, 2017
Publication
<1%

22 publisher.medfak.ni.ac.rs
Internet Source
<1%

23 "Methods", Vaccine, 20071101
Publication
<1%

24 biomed.news
Internet Source
<1%

25 Kelu Yang, Ya Gao, Yitong Cai, Ming Liu, Cuncun Lu, Junhua Zhang, Jinhui Tian. "Trends analysis of cancer topic of Cochrane systematic reviews: a bibliometric analysis", Research Square, 2019
Publication
<1%

26 Yanqiao Zhao, Ziping Zhang, Suimin Guo, Beibei Feng, Xiaoyu Zhao, Xueqiang Wang, Yuling Wang. "Bibliometric Analysis of Research Articles on Pain in the Elderly Published from 2000 to 2019", Journal of Pain Research, 2021
Publication
<1%

27	www.gecekitapligi.com<br>Internet Source	<1 %
28	Anja Kovanda, Ana Nyasha Zimani, Borut Peterlin. "How to design a national genomic project—a systematic review of active projects", Human Genomics, 2021<br>Publication	<1 %
29	Cécile Le Page, Jacqueline Chung, Kurosh Rahimi, Martin Köbel, Diane Provencher, Anne-Marie Mes-Masson. "Exploring the Clinical Impact of Predictive Biomarkers in Serous Ovarian Carcinomas", Current Drug Targets, 2020<br>Publication	<1 %
30	Joyjit Chatterjee, Nina Dethlefs. "Scientometric review of artificial intelligence for operations & maintenance of wind turbines: The past, present and future", Renewable and Sustainable Energy Reviews, 2021<br>Publication	<1 %
31	smw.ch<br>Internet Source	<1 %
32	Belinda W. C. Ommering, Floris M. van Blankenstein, Merel van Diepen, Friedo W. Dekker. "Academic Success Experiences: Promoting Research Motivation andSelf-	<1 %

Efficacy Beliefs among Medical Students", Teaching and Learning in Medicine, 2021
Publication

33	Submitted to Yildirim Beyazit Universitesi<br>Student Paper	<1 %
34	Submitted to An-Najah National University<br>Student Paper	<1 %
35	Lile Dong, Wenjuan Li, Luodan Yu, Lining Sun, Yu Chen, Guobin Hong. " Ultrasmall Ag Te Quantum Dots with Rapid Clearance for Amplified Computed Tomography Imaging and Augmented Photonic Tumor Hyperthermia ", ACS Applied Materials & Interfaces, 2020<br>Publication	<1 %
36	Yujia Dai, Kresna Hartandi, Zhiqin Ji, Asma A. Ahmed et al. " Discovery of -(4-(3-Amino-1 -indazol-4-yl)phenyl)- ‘-(2-fluoro-5-methylphenyl)urea (ABT-869), a 3-Aminoindazole-Based Orally Active Multitargeted Receptor Tyrosine Kinase Inhibitor ", Journal of Medicinal Chemistry, 2007<br>Publication	<1 %
37	res.mdpi.com<br>Internet Source	<1 %
38	www.mdpi.com<br>Internet Source	

<1%

39 Sam Weiss Evans. "Synthetic biology: Missing the point", Nature, 2014
Publication <1%

40 www.frontiersin.org
Internet Source <1%

41 Submitted to University of Birmingham
Student Paper <1%

42 Yichao Wan, Shengzhuo He, Wei Li, Zilong Tang. "Indazole Derivatives: Promising Anti-tumor Agents", Anti-Cancer Agents in Medicinal Chemistry, 2019
Publication <1%

43 Razieh Salehian, Mehdi Nasr Esfahani. "Therapeutic Challeng es of COVID-19 in a Patient Admitted to the Psychosomatic Ward: A Case Report", Iranian Journal of Psychiatry and Behavioral Sciences, 2021
Publication <1%

44 www.intechopen.com
Internet Source <1%

45 Jae Ryul Bae, Wook Ha Park, Dong Hoon Suh, Jae Hong No, Yong Beom Kim, Kidong Kim. "Role of limonin in anticancer effects of Evodia rutaecarpa on ovarian cancer cells", <1%

BMC Complementary Medicine and Therapies, 2020
Publication

46	www.nature.com<br>Internet Source	<1 %
47	Submitted to University of the West Indies<br>Student Paper	<1 %
48	lrd.yahooapis.com<br>Internet Source	<1 %
49	translational-medicine.biomedcentral.com<br>Internet Source	<1 %
50	Khalid El Bairi, Said Afqir, Mariam Amrani. "Is HE4 Superior over CA-125 in the Follow-up of Patients with Epithelial Ovarian Cancer?", Current Drug Targets, 2020<br>Publication	<1 %
51	Maricruz Anaya-Ruiz, Ana Karen Vincent, Martin Perez-Santos. "Cervical Cancer Trends in Mexico: Incidence, Mortality and Research Output", Asian Pacific Journal of Cancer Prevention, 2014<br>Publication	<1 %
52	ir.kagoshima-u.ac.jp<br>Internet Source	<1 %
53	www.semanticscholar.org<br>Internet Source	<1 %

54	Meriem Slaoui, Rachid Razine, Azeddine Ibrahimi, Mohammed Attaleb, Mohammed El Mzibri, Mariam Amrani. "Breast Cancer in Morocco: A Literature Review", Asian Pacific Journal of Cancer Prevention, 2014<br>Publication	<1 %
55	link.springer.com<br>Internet Source	<1 %
56	text.soe.ucsc.edu<br>Internet Source	<1 %
57	www.biomeddefinition.com<br>Internet Source	<1 %
58	"Illuminating Colorectal Cancer Genomics by Next-Generation Sequencing", Springer Science and Business Media LLC, 2020<br>Publication	<1 %
59	Danyang Chen, Ge Zhang, Jiahui Wang, Shiling Chen, Jingxuan Wang, Hao Nie, Zhouping Tang. "Mapping Trends in Moyamoya Angiopathy Research: A 10-Year Bibliometric and Visualization-Based Analyses of the Web of Science Core Collection (WoSCC)", Frontiers in Neurology, 2021<br>Publication	<1 %
60	Dörthe Brüggmann, Vanessa Handl, Doris Klingelhöfer, Jenny Jaque, David A Groneberg. "Congenital toxoplasmosis: an in-depth	<1 %

density-equalizing mapping analysis to explore its global research architecture", Parasites & Vectors, 2015
Publication

---

61 Kaihua Chen, Yi Zhang, Xiaolan Fu. "International research collaboration: An emerging domain of innovation studies?", Research Policy, 2018
Publication
<1%

---

62 Khalid El Bairi, Mariam Amrani. "The Power of Biomarkers in Transforming Patients Care in Gynecologic Oncology", Current Drug Targets, 2020
Publication
<1%

---

63 Nadin Younes, Hatem Zayed. "Genetic epidemiology of ovarian cancer in the 22 Arab countries: A systematic review", Gene, 2019
Publication
<1%

---

64 doaj.org
Internet Source
<1%

---

65 ecancer.org
Internet Source
<1%

---

66 pubmed.ncbi.nlm.nih.gov
Internet Source
<1%

---

67 worldwidescience.org
Internet Source
<1%

68	www.thieme-connect.com<br>Internet Source	<1%
69	Ashton Barnett-Vanes, Guiyi Ho, Timothy M Cox. "Clinician-scientist MB/PhD training in the UK: a nationwide survey of medical school policy", BMJ Open, 2015<br>Publication	<1%
70	K. C. Garg, S. Kumar. "Contribution of Indian women scholars to Indian output in bibliometrics/scientometrics and the pattern of co-authorship and collaboration", COLLNET Journal of Scientometrics and Information Management, 2019<br>Publication	<1%
71	Elżbieta Bielecka, Elżbieta Burek. "Spatial data quality and uncertainty publication patterns and trends by bibliometric analysis", Open Geosciences, 2019<br>Publication	<1%
72	bmccancer.biomedcentral.com<br>Internet Source	<1%

Exclude quotes	Off	Exclude matches	Off
Exclude bibliography	Off		
